# Changes in Cannabis Consumption During the Global COVID-19 Lockdown: The International COVISTRESS Study

**DOI:** 10.3389/fpsyt.2021.689634

**Published:** 2021-11-11

**Authors:** Juliette Salles, Antoine Yrondi, Fouad Marhar, Nicolas Andant, Raimundo Avilés Dorlhiac, Binh Quach, Jiao Jiao, Samuel Antunes, Ukadike Chris Ugbolue, Julien Guegan, Karine Rouffiac, Bruno Pereira, The COVISTRESS Network, Nicolas Andant, Maëlys Clinchamps, Frederic Dutheil

**Affiliations:** ^1^University Hospital of Toulouse, CHU Toulouse, Department of Psychiatry, Infinity (Toulouse Institute for Infectious and Inflammatory Diseases), INSERM UMR1291, CNRS UMR5051, Université Toulouse III, Toulouse, France; ^2^University Hospital of Toulouse, CHU Toulouse, Department of Psychiatry, Inserm Toulouse NeuroImaging Center, ToNIC, Toulouse, France; ^3^Université Clermont Auvergne, CNRS, LaPSCo, Physiological and Psychosocial Stress F-63000 Clermont-Ferrand, France University Hospital of Toulouse, Department of Anaesthesiology and Critical Care, Toulouse, France; ^4^University Hospital of Clermont-Ferrand, CHU Clermont-Ferrand, DRCI, Biostatistics Unit, Clermont-Ferrand, France; ^5^Universidad Finis-Terrae, El-Carmen, Hospital Dr. Luis-Valentìn-Ferrada, Obstetrics and Gynecology, Maipù, Chile; ^6^Sport and Physical Education, Hong Kong Baptist University, Hong Kong, Hong Kong SAR, China; ^7^Ordem dos Psicólogos Portugueses, ISPA-Instituto Universitário, Lisbon, Portugal; ^8^University of the West of Scotland, Institute for Clinical Exercise & Health Science, School of Health and Life Sciences, Glasgow, United Kingdom; ^9^Université Clermont Auvergne, CNRS, LaPSCo, Catech, Clermont-Ferrand, France; ^10^University Hospital of Clermont-Ferrand, CHU Clermont-Ferrand, Preventive and Occupational Medicine, Clermont-Ferrand, France; ^11^Université Clermont Auvergne, CNRS, LaPSCo, Physiological and Psychosocial Stress, University Hospital of Clermont-Ferrand, CHU Clermont-Ferrand, Preventive and Occupational Medicine, WittyFit, Clermont-Ferrand, France

**Keywords:** cannabis (marijuana), COVID-19, addiction, lockdown, tobacco

## Abstract

**Introduction:** COVID-19 lockdown measures have been sources of both potential stress and possible psychological and addiction complications. A lack of activity and isolation during lockdown are among the factors thought to be behind the growth in the use of psychoactive substances and worsening addictive behaviors. Previous studies on the pandemic have attested to an increase in alcohol consumption during lockdowns. Likewise, data suggest there has also been a rise in the use of cannabis, although it is unclear how this is affected by external factors. Our study used quantitative data collected from an international population to evaluate changes in cannabis consumption during the lockdown period between March and October, 2020. We also compared users and non-users of the drug in relation to: (1) socio-demographic differences, (2) emotional experiences, and (3) the information available and the degree of approval of lockdown measures.

**Methods:** An online self-report questionnaire concerning the lockdown was widely disseminated around the globe. Data was collected on sociodemographics and how the rules imposed had influenced the use of cannabis and concerns about health, the economic impact of the measures and the approach taken by government(s).

**Results:** One hundred eighty two respondents consumed cannabis before the lockdown vs. 199 thereafter. The mean cannabis consumption fell from 13 joints per week pre-lockdown to 9.75 after it (*p* < 0.001). Forty-nine respondents stopped using cannabis at all and 66 admitted to starting to do so. The cannabis users were: less satisfied with government measures; less worried about their health; more concerned about the impact of COVID-19 on the economy and their career; and more frightened of becoming infected in public areas. The risk factors for cannabis use were: age (OR = 0.96); concern for physical health (OR = 0.98); tobacco (OR = 1.1) and alcohol consumption during lockdown (OR = 1.1); the pre-lockdown anger level (OR = 1.01); and feelings of boredom during the restrictions (OR = 1.1).

**Conclusion:** In a specific sub-population, the COVID-19 lockdown brought about either an end to the consumption of cannabis or new use of the drug. The main risk factors for cannabis use were: a lower age, co-addictions and high levels of emotions.

## Introduction

The COVID-19 pandemic started in the Chinese city of Wuhan in December 2019 and subsequently spread globally (1). Then without a vaccine or any effective treatments, governments worldwide responded by implementing lockdown measures that aimed to limit the spread of the virus by restricting population movement and social contact (2). The introduction and economic consequences of these measures and uncertainty about the course of the epidemic have been sources of stress and social isolation (3).

In many countries, cannabis is one of the psychotropic drugs consumed the most (4), with research into its use linking it to addictive behaviors (5). Taking psychoactive substances (and consequential addictive behaviors) can be a coping mechanism for individuals experiencing stress or negative moods (6), as well as for those who are unable to face difficult situations and, as a result, reduce their social interactions (7–11). Substance use and addictive behaviors may therefore be seen as a remedy for boredom (12, 13) and social isolation (14).

Cannabis can also be used to reduce emotional reactivity. Indeed, its consumption is associated with the activation of cannabinoid receptors that mediate the neural processes underlying emotional regulation and stress responsivity (15). Moreover, the endocannabinoid system also counteracts the neurochemicals involved in the use of other substances, including those playing a part in emotional regulation. The signals of cannabinoid receptors, for example, might counteract the neurochemical imbalance associated with alcohol withdrawal (16).

These factors are all worthy of consideration when examining the impact of COVID-19 lockdowns on cannabis use. Previous studies have provided interesting data and attested to the effects of these lockdowns on use of the drug, with some of them [e.g., Rolland et al. (17)] reporting an increase in cannabis consumption in lockdown periods. Unfortunately, however, that study was only conducted among a French population and does not analyze changes in consumption levels. In Belgium, meanwhile, Vanderbruggen et al. (11) found no statistically significant differences between the number of joints smoked per day before and during lockdown. Nevertheless, the value of this study is limited by its recruitment of a higher than ideal proportion of educated women and the overrepresentation of healthcare workers. Conversely, a study by Cousijn et al. (18) described an increase in lockdown cannabis use, but only involved a Dutch population. Finally, a survey by the European Monitoring Centre for Drugs and Drug Addiction (EMCDDA) found that occasional users had either stopped, or at least reduced, their consumption of the drug during lockdown. The levels of consumption by heavy users had, however, increased, with the drug employed to relieve anxiety and boredom (19). Nevertheless, this research mainly involved young respondents, with an average age of 29-years, while participants from Estonia, Spain, Italy and Finland accounted for 50% of the study's population, the majority of which was male (19).

Emotions play a critical role in the use of substances. Indeed, impairments in the regulation of emotion contribute to the development and severity of substance use disorders (SUDs) (and addictive behaviors), and are also associated with neurobiological damage consisting of increased amygdala and insula activation (20) and a weakening of the capacity to recognize emotions (alexithymia) (21, 22). Moreover, substances can be used to regulate emotion. Animal models, for example, have suggested that a moderate intake of alcohol reduces emotionality and facilitates adaptive responses and problem solving (23, 24).

The COVID-19 pandemic induced emotional states that led to people becoming less happy and more anxious, fearful, and angry (25). In addition, studies have reported an increase in alcohol consumption during lockdowns (26, 27), which may be consistent with the theory that substances are used to regulate emotions. Consequently, it could be hypothesized that the changes induced by lockdown measures may have affected the population's use of drugs, with those suffering from an SUD and/or behavioral addictions particularly vulnerable (28) due to the increase in the consequences of, and behavior caused by, consumption (e.g., alcohol could impact emotional and behavioral reactivity) (23, 24).

Despite their interesting results, the aforementioned studies (11, 17–19) provide limited data on the association between emotional changes and cannabis consumption, in particular on the role played by these emotions in the use of substances. In order to remedy this, our research uses quantitative data collected from a population recruited internationally to evaluate changes in the consumption of cannabis during lockdown. It also compares users and non-users of the drug in relation to: (1) sociodemographic differences; (2) emotional experiences; and (3) the information available on and degree of approval of measures introduced during the lockdown period between March and October, 2020.

## Method

### Study Design

We conducted an international, prospective, observational study of a general population in the period March to October, 2020 (hereafter: the lockdown). A computerized, anonymous questionnaire, translated into ten languages, was used for this purpose. The main academic partners in this research form “The COVISTRESS network” and are named at the start of the paper. This list of contributors to the project is regularly updated on the website https://covistress.org/contacts.html and currently comprises 21 main partner-countries and 70 researchers, across five continents. The questionnaire that forms the basis of the study was distributed electronically to facilitate its dissemination. The research has been given the required ethics approval and is registered on ClinicalTrials.gov (NCT04538586) (3, 29).

### Inclusion Criteria

The international COVISTRESS network was used to distribute the questionnaire to respondents from the general population, with no country, gender, occupation or disease distinctions made.

### Outcomes

We evaluated the consumption of cannabis before and after the introduction of lockdown measures. The primary outcome, i.e., cannabis consumption, was measured based on the number of joints smoked per week. To this end, we asked a single question [how many “joints” (of cannabis) do you smoke per week?] twice (before the pandemic/during the first lockdown).

The secondary outcomes were: sociodemographics (age, sex, level of education, country of origin); alcohol consumption, based on the number of drinks per week; tobacco consumption, i.e., the number of cigarettes smoked a day; worries (about health, the impact of COVID-19 on the economy and the healthcare system); the information available to the respondents and the degree of approval of the measures introduced during the lockdown (distrust of government restrictions or level of confidence); and emotions (peaceful and angry, sad and happy, calm and excited, busy and bored). Sociodemographic data were obtained via multiple-choice questions. Worries and emotions (as above) were retrieved using visual analogue scales (VASs), i.e., a non-calibrated horizontal line ranging from a minimum (0) to a maximum (100) (30–32).

### Statistical Analyses

The analyses of the quantitative data were conducted using the means and standard deviations or the median and the interquartiles based on the distribution of the responses to the questionnaire. Parametric tests (*T*-test) were employed to perform the comparative analyses. The qualitative variables were examined with the Chi-squared test. The significance threshold was set at *p* < 0.05. Pearson correlations were used to measure the associations between the variables. The links between cannabis consumption during the lockdown period and the variables employed in the questionnaire were evaluated using multinomial logistic regression. The responses determined by the comparative analyses to be significantly different between the cannabis users and non-users were then introduced into the model (33). The sociodemographic variables (gender, age, sociodemographic status, level of education, country of origin) were integrated in the analysis as confounding factors. The statistical significance threshold was set at *p* < 0.05. The analyses were carried out with the Jamovi statistical program, version 1.2 (the Jamovi project, 2020) and the R studio software package, version 3.6 (34).

## Results

### Sociodemographic Data

**Figure 1:**
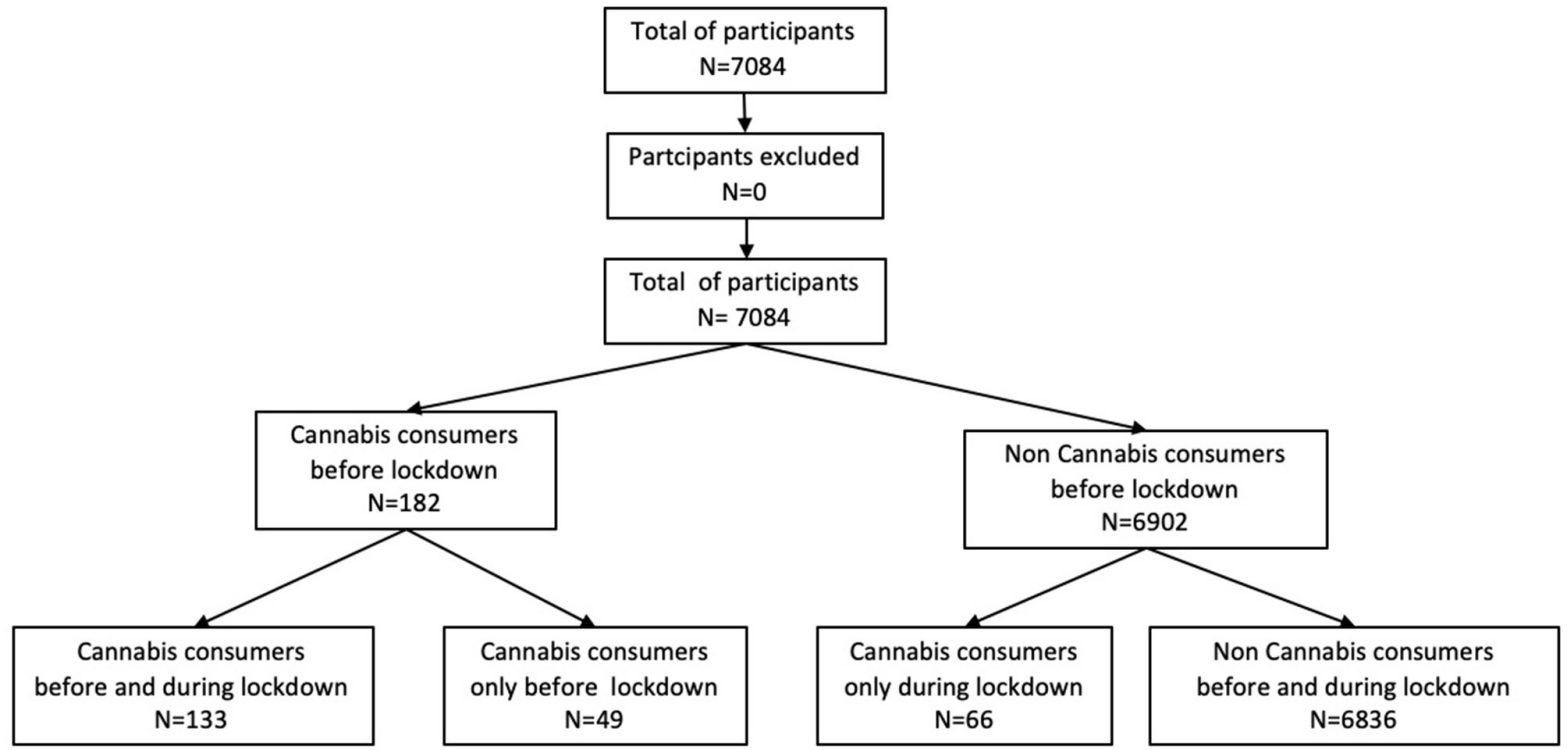
Flow chart on the recruitment of participants.

A total of 7,084 people answered the survey questions and were included in the study (Figure 1). Of these respondents, 4,875 (69%) were female and 2,209 (31%) male. The mean ± standard deviation (SD) age was 42.3 ± 13.3 years. The participants lived in 57 countries (6,572 in Europe, 218 in Asia, 167 in America, 57 in Africa, four in Oceania and 65 non-specified). In terms of education: 667 (9%) were educated to a level below a bachelor's degree; 907 (13%) had the equivalent of such a degree; 2,645 (37%) had a license degree; 1,958 (28%) had a master's degree; and 907 (13%) were educated above this level.

### Cannabis Consumption

Prior to and during the lockdown, 182 (2.5%) and 199 (2.8%) respondents, respectively, were cannabis users. Men comprised 52% of the pre-lockdown consumers of the drug. The mean ± SD age of the cannabis-using respondents was 35 ± 12.3 years and they had an educational level of 3.9 years (± 1.1). The mean number of joints smoked per week prior to the lockdown was 13 ± 4.1 (median = 13) vs. 9.75 ± 7.1 (median = 13) during it. This difference was significant (*p* < 0.001). The differential between the number of cannabis users before and after the lockdown is due to 49 respondents who ended their cannabis consumption and 66 non-users who started to consume it. The details of the cannabis use of each group, including their levels of consumption of tobacco and alcohol, are set out in Table 1.

**Table 1:** Presentation of the data for the overall group and the subgroups of cannabis users and non-users; *n* (%): number of individuals (percentage) or mean ± standard-deviation.

	**Cannabis users**					**Non-users before and during lockdown**	**Users** **vs. non-users during lockdown**
	**Before lockdown**	**Only before lockdown**	**During lockdown (*****n*** **= 199)**				
			**Before & during lockdown**	**Only during lockdown**	**Total**		
	***n* = 182**	***n* = 49**	***n* = 133**	***n* = 66**	***n* = 199**	***n* = 6,836**	***P*-value**
Gender, *n* male (%)	95 (52%)	25 (52%)	69 (52%)	46 (70%)	115 (61%)	6,836 (69%)	0.61
Age in years	35 ± 12.3	33.8 ± 11.5	35.5 ± 12.6	39.7 ± 13.5	37.6 ± 13.1	42.6 ± 13.3	<0.001^*^
Level of education, year postgraduate?	3.9 ± 1.1	3.8 ± 1.2	4 ± 1.1	4.3 ± 1.2	4.15 ± 1.1	4.2 ± 1.2	0.29
Worries about health, VAS (0 to 100)	48.2 ± 30.7	48.6 ± 29.4	48.1 ± 31.3	44.7 ± 33.6	46.4 ± 32.4	54.1 ± 30.4	0.03^*^
Stress of covid, VAS (0 to 100)	55.5 ± 31.2	55.4 ± 30.9	55.6 ± 31.4	57.8 ± 30	56.7 ± 30.7	57.7 ± 29.9	0.53
Fatigue, VAS (0 to 100)	53.4 ± 31.4	55.0 ± 31.6	52.8 ± 31.5	55.0 ± 32.2	53.9 ± 31.9	51.0 ± 31.8	0.31
Anxiety-fear, VAS (0–100)	53.3 ± 30.9	58 ± 32.4	51.5 ± 30.3	52.4 ± 30.4	51.95 ± 30.4	50.9 ± 30.6	0.79
Good mood, VAS (0–100)	48 ± 29.4	44.3 ± 31.7	49.4 ± 28.5	50.7 ± 31.2	50.05 ± 29.9	53.1 ± 27	0.12
Worries about economic impact, VAS (0–100)	75.0 ± 26	68.9 ± 33.3	77.2 ± 22.5	78 ± 19.8	77.6 ± 21.2	76.9 ± 22.4	0.71
Worries re impact on healthcare system, VAS (0–100)	72.2 ± 27	76.1 ± 25.8	70.7 ± 27.4	66.8 ± 23.4	68.75 ± 25.4	69.2 ± 25	0.87
Satisfaction with government measures, VAS (0–100)	35.9 ± 30.4	29.1 ± 29.8	38.3 ± 30.4	47.5 ± 32.3	42.9 ± 31.4	47.8 ± 30.5	0.005^*^
Satisfaction with measures for businesses, VAS (0–100)	65.0 ± 31.1	65.5 ± 27.9	64.9 ± 32.4	66.2 ± 32.9	65.55 ± 32.7	66.2 ± 29	0.13
Smoking, *n* cigarettes/day							
Before lockdown	9.2 ± 6.4	10.9 ± 6.4	8.6 ± 6.3	4.3 ± 5.8	6.5 ± 6.1	2.3 ± 5.3	<0.001^*^
During lockdown	9.6 ± 7.1	11.3 ± 7.2	9.0 ± 7.0	5.4 ± 6.8	7.2 ± 6.9	2.3 ± 5.4	<0.001^*^
Alcohol, *n* units/week							
Before lockdown	9.3 ± 5.9	8.2 ± 6.4	9.7 ± 5.7	9.4 ± 5.3	9.6 ± 5.5	7.5 ± 6.2	<0.001^*^
During lockdown	8.9 ± 6.3	7.2 ± 6.6	9.5 ± 6.1	9.9 ± 4.9	9.7 ± 5.5	7.0 ± 6.2	<0.001^*^
Cannabis, *n* of joints/week							
Before lockdown	13.0 ± 4.1	13.2 ± 3.9	13.0 ± 4.1	0.0 ± 0.0	13 ± 2.1	0.0 ± 0.0	<0.001^*^
During lockdown	9.7 ± 7.1	0.0 ± 0.0	13.3 ± 4.7	12 ± 2.2	12.7 ± 3.5	0.0 ± 0.0	<0.001^*^
Peaceful/angry, VAS from peaceful (0) to angry (100)							
Before lockdown	42.6 ± 25	41.1 ± 24.1	43.1 ± 25.4	43.1 ± 23.7	43.1 ± 24.5	37.9 ± 22.7	0.02^*^
During lockdown	60.2 ± 27.1	64.8 ± 24.6	58.5 ± 27.9	58.4 ± 25.0	58.5 ± 26.4	54.5 ± 25.5	0.09
Sad-happy, VAS from sad (0) to happy (100)							
Before lockdown	65.7 ± 23.4	63.3 ± 24.9	66.5 ± 22.9	64.8 ± 19.8	65.7 ± 21.35	68.6 ± 21.0	0.03^*^
During lockdown	42.3 ± 26.2	35.8 ± 26.8	44.6 ± 25.6	47.1 ± 24.1	45.9 ± 24.9	47.1 ± 24.8	0.41
Calm-excited, VAS from calm (0) to excited (100)							
Before lockdown	48.0 ± 27.8	51.5 ± 26.1	46.7 ± 28.3	55.6 ± 22	51.2 ± 25.2	43.5 ± 25.0	0.03^*^
During lockdown	49.3 ± 27.4	52.1 ± 28.1	48.4 ± 27.2	51.1 ± 26.3	49.8 ± 26.8	46.7 ± 24.8	0.24
Busy-bored, VAS from busy (0) to bored (100)							
Before lockdown	22.3 ± 22.5	24.9 ± 24.1	21.3 ± 21.9	20.8 ± 22.6	21.1 ± 22.3	19.1 ± 19.2	<0.001^*^
During lockdown	51.3 ± 31.2	55.7 ± 28	49.6 ± 30.9	52.9 ± 30.4	51.2 ± 30.7	40.4 ± 30.6	<0.001^*^

*Bold value indicated the p-value < 0.05*.

* *p < 0.05*.

**Figure 2:**
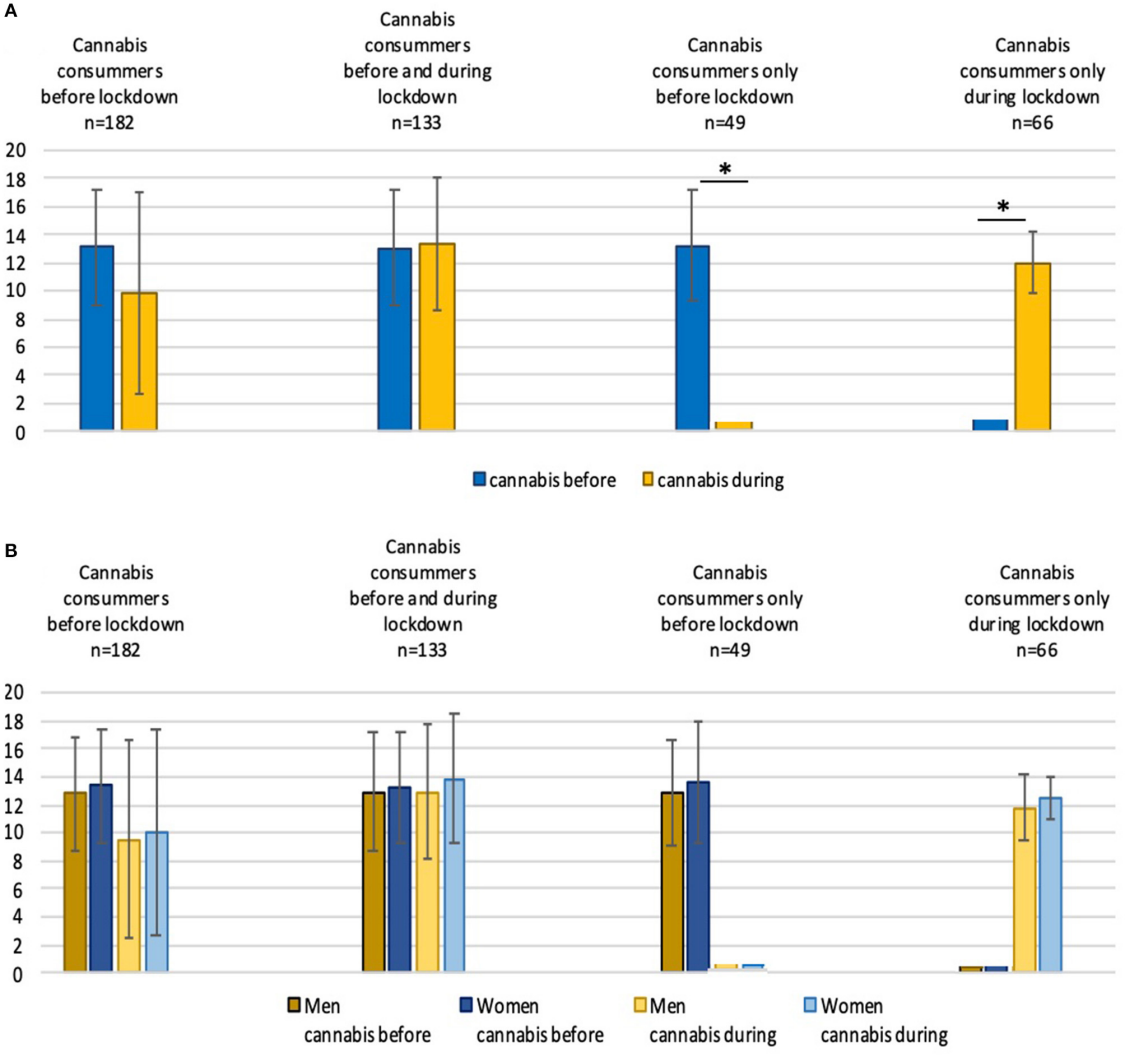
**(A)** Comparison of the consumption of cannabis before and during the lockdown period in the overall group of cannabis users and the three subgroups. **(B)** Comparison of the consumption of cannabis before and during the lockdown in men and women. The ordinate represents the number of joints consumed per week *p < 0.05.

The mean cannabis consumption before the lockdown was 12.8 joints per week ± 4.0 (median = 13) for the male respondents and 13.3 ± 4.1 (median = 13) for the female (*p* = 0.21). These amounts during the lockdown were 9.5 joints per week ± 7.0 (median = 8) for the men and 10 ± 4.1 (median = 10) for the women (*p* = 0.13). Figure 2 shows the changes in consumption of each group and the effects these changes had on the male and female participants.

### Comparison of Cannabis Users and Non-users During the Lockdown

The lockdown cannabis users (*n* = 199) were 37.6 years old ± 13.1 vs. 42.6 ± 13.3 years for the non-users (*p* < 0.001). The former were: less satisfied with their government's restrictions (*p* < 0.05); less concerned about their health (*p* = 0.03); more concerned about the impact of COVID-19 on the economy (*p* < 0.05) and their career (*p* < 0.05); and more worried about catching the disease in public areas (*p* = 0.04). Pre-lockdown, the cannabis users consumed, on average, more alcohol (9.6 glasses per week ± 5.5) than the non-users (7.5 glasses per week ± 6.2) (*p* < 0.001). This pattern continued during the lockdown, with the cannabis users drinking more than the non-users: 9.7 units per week ± 5.5 vs. 7.0 ± 6.2. This difference is significant (*p* < 0.001). Similarly, in the pre-lockdown period, the cannabis users consumed, on average, more tobacco than the non-users−6.5 cigarettes per day ± 6.1 vs. 2.3 per day ± 5.3 (*p* < 0.001), respectively. This continued during the lockdown, with the cannabis users smoking 7.2 cigarettes per day ± 6.9 and the non-users 2.3 per day ± 5.4. This difference is also significant (*p* < 0.001). Boredom levels were higher in the cannabis-user group both before and during the lockdown: 21.1 ± 22.3 vs. 19.1 ± 19.2 (*p* < 0.001), respectively; these figures for the non-users were 51.2 ± 30.7 vs. 40.4 ± 30.6 (*p* < 0.001). The study's other parameters did not reveal any further differences between the groups, as reported in Table 1.

### Multivariate Analysis

**Figure 3:**
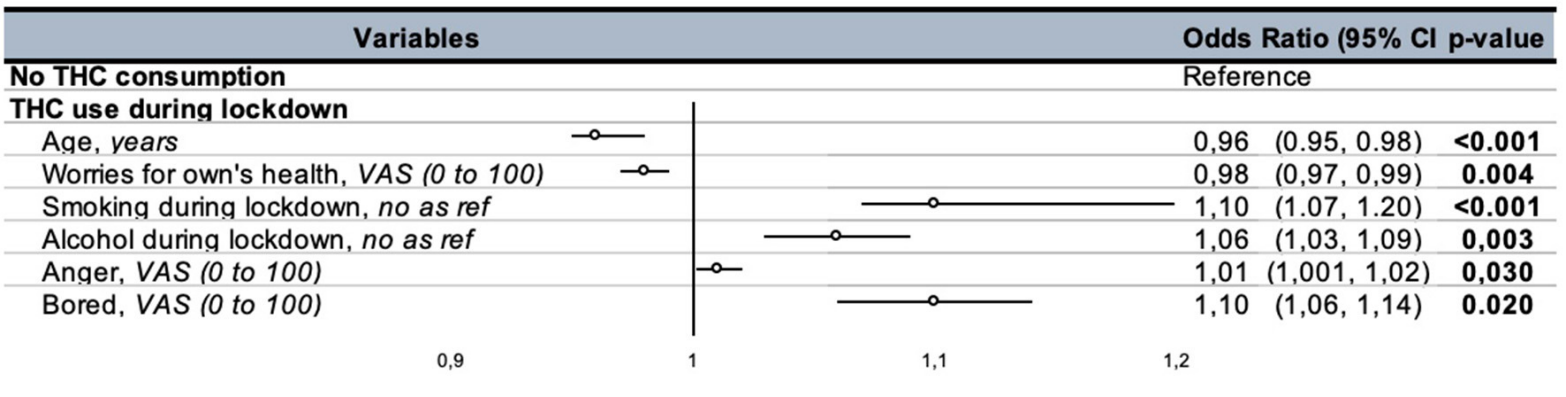
Results of the regression analysis of the use of cannabis during the lockdown. VAS, visual analogue scale.

The factors that had a significant association with cannabis consumption during the lockdown were: age (OR = 0.96, 95% CI: 0.95–0.98, *p* < 0.001); concern for physical health (OR = 0.98, 95% CI: 0.97–0.99, *p* = 0.004); tobacco consumption during the lockdown (OR = 1.10, 95% CI: 1.07–1.20, *p* < 0.001); alcohol consumption in the lockdown (OR = 1.06, 95% CI: 1.03–1.09, *p* = 0.003); the level of anger pre-lockdown (OR = 1.01, 95% CI: 1.001–1.017, *p* = 0.03); and feeling bored during the lockdown (OR = 1.10, 95% CI: 1.06–1.14, *p* = 0.02) (Figure 3).

The factors significantly associated with ending the consumption of cannabis were: smoking tobacco pre-lockdown (OR = 1.1, 95% CI: 1.01–1.14, *p* = 0.01) and concern about the economic impact of the crisis (OR = 0.98, 95% CI: 0.96–0.99, *p* = 0.01). The elements linked to new cannabis use were: consuming alcohol before the lockdown (OR = 1.05, 95% CI: 1.009–1.09, *p* = 0.01) and feeling bored during it (OR = 1.01, 95% CI: 1.003–1.02, *p* = 0.006). Concern for health was negatively associated with starting to consume the drug (OR = 0.98, 95% CI: 0.97–0.99, *p* = 0.005).

## Discussion

Our study aimed to document the impact of the COVID-19 lockdown measures in force from March to October 2020 on cannabis consumption in an international population. The study's results revealed that 2.5–2.8% of the respondents were cannabis users, which is consistent with such data globally (35). The factor most associated with cannabis use during the lockdown period was the consumption of other substances (tobacco and/or alcohol). The cannabis users were also younger in age, less concerned about their health, experienced more angry feelings pre-lockdown and were more bored during it.

The results also revealed that the cannabis-using group had greater distrust of government-imposed measures. A link between the degree of suspicion of politics and cannabis consumption has already been described in the literature (36). The low level of confidence in this association in our study could be partially explained by the existence of a link between addictive behavior and antisocial-personality traits (37, 38), although we did not collect any data that would enable this hypothesis to be accepted or rejected.

Our cannabis users reported being more worried about the impact of the pandemic on their career and the economy more generally. The association between such a concern and cannabis consumption reinforces the view that occupational physicians have an important role to play in the prevention and management of addictive behavior; indeed, data is already available on opportunities for motivational management in the workplace (39).

The part played by the environment is important in the development of addictive behaviors, which are defined on the basis of a bio-psychosocial approach (40). The data in the literature reveal a link between social isolation and the risk of developing addictions (41–43). Consequently, in the context of social isolation associated with the lockdown, we expected to see an increase in the amounts of cannabis consumed. In fact, there was a significant reduction in the cannabis-using group. We hypothesized that this could partly be due to less access to the drug, but the reduction was not homogenous, being explained by the actions of a sub-group of 49 individuals who stopped using cannabis at all; meanwhile, the quantities smoked by those who continued to consume the drug remained stable. These outcomes indicate that levels of vulnerability to the effects of lockdown measures differ, with some cannabis consumers having a positive experience. Moreover, the lockdown measures may have affected the availability of cannabis; indeed, social distancing might reasonably be expected to disrupt established methods for supplying and distributing the drug. Nonetheless, some of our users moved to online purchasing (44), while others may not have respected the restrictions as intended. This is, however, only a hypothesis, and its premises could be differentially explained by underlying factors like a change in income levels and/or the use of other/stronger drugs. Differences in the lockdown legislation in force in the countries where the respondents live may also be a factor. Indeed, the legality of cannabis use in some areas may have limited the effects of the lockdown measures on the availability of the drug.

Other studies have demonstrated that bringing an end to cannabis use can have an effect on the consumption of other substances. Consistent with this, our research identified an increase in alcohol use in particular (45, 46). However, there were no changes in the amounts of alcohol or tobacco consumed by the group that stopped using the drug at all. Tobacco use pre-lockdown was associated with an end to cannabis consumption during it: 47 of our cannabis users (26%) did not consume tobacco before the lockdown, and it was these individuals who were less likely to stop their use of the drug in the relevant period. Concern about the economic impact of the health crisis was also a risk factor for continued cannabis use.

Conversely, a sub-group of 66 respondents started to smoke cannabis during the lockdown, corresponding to 1% of those who did not use the drug before it. Drinking alcohol pre-lockdown and feeling bored during it appeared to be risk factors for this. Boredom certainly seems to be associated with use of the drug, but may not be the only explanation.

Despite these interesting results, our study has some limitations. A major issue relates to our lack of screening for the duration of the lockdown, changes in income (before and after lockdown), and the use of addictive drugs other than tobacco, alcohol and cannabis. These factors may therefore also account for our findings. Specifically, controlling for the lockdown duration (as a covariate of non-interest) is important. In addition, the failure to consider the impact of a reduced income and the use of stronger drugs is an important limitation, as these factors may account for why some in the cannabis-user group stopped using the drug during the lockdown. A further limitation relates to the study's design, namely an online survey, which may induce selection bias. Moreover, the design was used to collect data with which to establish associations, but did not permit the identification of causal links. A “multiple time-point" prospective observational study would be valuable for this purpose. Additionally, the natural turnover between being a cannabis user or not is impossible to assess, and reaching robust conclusions about the effects of the lockdown thus warrants further research. Another limitation concerns the fact that the survey was not validated, although most of the other studies on cannabis use ask a comparable question about consumption (47, 48). Similarly, our questionnaire did not produce data about addictive behavior. Indeed, the quantities of cannabis consumed by our respondents did not support such a diagnosis. It would therefore have been interesting to collect data relating to the DSM-5 criteria (49) in order to better characterize our population. Nevertheless, our survey was addressed to the general population and was intended to produce a wide variety of participants. This meant that decisions had to be made about what questions to include in order to limit the amount of time required to answer them. Quality assurance of the COVISTRESS questionnaire was ensured by the fact that only one questionnaire was submitted per IP address. However, it is possible that the same participant submitted several surveys from different IP addresses. Moreover, the study had a greater proportion of females to males, but, unfortunately, it was not possible to control for this gender imbalance. Finally, all of our reported ORs are very close to 1. Even though the analysis did achieve statistical significance, the clinical impact should be confirmed in future research.

## Conclusion

Our study reveals changes in cannabis consumption during the COVID-19 lockdowns imposed from March to October, 2020. In particular, it highlights the existence of a specific sub-population for whom the lockdown brought about either the end to or the start of cannabis consumption. The results show that cannabis users can be characterized as having features specific to them in terms of their concerns about public policies and work stress. Acknowledging this could lead to a better provision of information and the use of targeted support.

## Data Availability Statement

The raw data supporting the conclusions of this article will be made available by the authors, without undue reservation.

## Ethics Statement

The studies involving human participants were reviewed and approved by National CPP. The patients/participants provided their written informed consent to participate in this study.

## The Covistress Network

The members of the research group are: Nicolas Andant, Maélys Clinchamps, Stéphanie Mestres, Cécile Miele, Valentin Navel, Lénise Parreira, Bruno Pereira, Karine Rouffiac—all from CHU Clermont-Ferrand, France; Yves Boirie, Jean-Baptiste Bouillon-Minois, Martine Duclos, Maria Livia Fantini, Jeannot Schmidt, Stéphanie Tubert-Jeannin—all from Université Clermont Auvergne/CHU Clermont-Ferrand, France; Mickael Berthon, Pierre Chausse, Michael Dambrun, Sylvie Droit-Volet, Julien Guegan, Serge Guimond, Laurie Mondillon, Armelle Nugier, Pascal Huguet—all from Université Clermont Auvergne, CNRS, LAPSCO, France; Samuel Dewavrin—WittyFit, France; Fouad Marhar—CHU Toulouse, France; Geraldine Naughton, Amanda Benson—Swinburne University, Australia; Claus Lamm—University of Vienna, Austria; Karen Gbaglo—Ministery of Health, Benin; Vicky Drapeau—Université de Laval, Canada; Raimundo Avilés Dorlhiac—Universidad Finis Terrae, Chile; Benjamin Bustos—Universidad de Los Andes, Chile; Gu Yaodong—Ningbo University, China; Haifeng Zhang—Hebei Normal University, China; Peter Dieckmann—Copenhagen Academy for Medical Education and Simulation (CAMES), Denmark; Julien Baker, Binh Quach, Jiao Jiao, Yanping Duan, Gemma Gao, Wendy Y J Huang, Ka Lai Kelly Lau, Chun-Qing Zhang—all from Hong Kong Baptist University, China; Hijrah Nasir, Indonesia; Perluigi Cocco, Rosamaria Lecca, Monica Puligheddu, Michela Figorilli—all from Università di Cagliari, Italia; Morteza Charkhabi, Reza Bagheri—University of Isfahan, Iran; Daniela Pfabigan—University of Oslo, Norway; Peter Dieckmann—University of Stavanger, Norway; Samuel Antunes, David Neto, Pedro Almeida—all from Ordem dos Psicólogos Portugueses, ISPA-Instituto Universitário, Portugal; Maria João Gouveia—ISPA-Instituto Universitário, Portugal; Pedro Quinteiro—William James Center for Research, ISPA-Instituto Universitário; Constanta Urzeala—UNEFS, Romania; Benoit Dubuis—UNIGE, Switzerland; Juliette Lemaignen—Fondation INARTIS, Switzerland; Andy Liu—University of Taipei, Taiwan; Foued Saadaoui—King Abdulaziz University, Tunisia; Ukadike Chris Ugbolue—University of the West of Scotland, United Kingdom; Keri Kulik—Indiana University of Pennsylvania, USA; Kuan-chou Chen—National Taiwan University of Sport, Department of Sport Management, Taiwan.

## Author Contributions

JS and AY performed statistical analysis. JS, AY, FM, and FD wrote the article. NA, RD, BQ, JJ, SA, UU, JG, KR, BP, The COVISTRESS Network, MC, and FD designed the study and recruited the participants. FD coordinated the study. All authors contributed to the article and approved the submitted version.

## Conflict of Interest

The authors declare that the research was conducted in the absence of any commercial or financial relationships that could be construed as a potential conflict of interest.

## Publisher's Note

All claims expressed in this article are solely those of the authors and do not necessarily represent those of their affiliated organizations, or those of the publisher, the editors and the reviewers. Any product that may be evaluated in this article, or claim that may be made by its manufacturer, is not guaranteed or endorsed by the publisher.
